# Nutritional Impact of High-Dose Melphalan in Autologous Stem Cell Transplantation

**DOI:** 10.7759/cureus.105679

**Published:** 2026-03-23

**Authors:** Yasmine El Ouafa, Monsif FADI, Houda Benrahma, Nouama Bouanani

**Affiliations:** 1 Center for Doctoral Studies (CeDoc), University Mohammed VI of Health Sciences (UM6SS), Casablanca, MAR; 2 Interdisciplinary Laboratory of Biotechnology and Health, University Mohammed VI of Health Sciences (UM6SS), Casablanca, MAR; 3 Oncopathology, Cancer Biology, and Environment Laboratory, Mohammed VI Faculty of Medicine, Center for Doctoral Studies (Cedoc), Mohammed VI University of Health and Sciences (UM6SS), Casablanca, MAR; 4 Medicine, Mohammed VI University of Health and Sciences (UM6SS), Casablanca, MAR; 5 Hematology, Faculty of Medicine, Mohammed VI University of Health and Sciences (UM6SS), Casablanca, MAR

**Keywords:** gastrointes-tinal symptoms, hematological malignancies, hematopoietic stem cell transplant, screening of nutritional status, high - dose melphalan

## Abstract

High-dose melphalan is the standard conditioning treatment for patients undergoing autologous hematopoietic stem cell transplantation for hematological malignancies. Although effective, it often causes significant gastrointestinal side effects, such as mucositis, nausea, vomiting, and diarrhea, which can quickly affect patients’ nutrition, hydration, and electrolyte balance, leading to acute malnutrition. This nutritional decline increases the risk of infections, prolongs hospital stays, and can worsen short-term recovery. Routine nutritional assessment and early support are not consistently implemented across clinical settings, leading to variability in patient care. This editorial discusses how melphalan-related digestive toxicity affects patients’ nutritional status, reviews evidence of its clinical impact, and highlights the importance of regular nutritional monitoring and early intervention. Collaboration among hematologists, dietitians, nurses, and pharmacists, along with standardized nutritional protocols in transplant units, is crucial to help patients tolerate treatment better, recover faster, and improve overall outcomes.

## Editorial

High-dose melphalan (HDM) remains a cornerstone conditioning regimen for patients undergoing autologous hematopoietic stem cell transplantation (HSCT) for hematological malignancies. While this strategy has substantially improved disease control and transplant outcomes, it is frequently associated with significant gastrointestinal (GI) toxicity that can adversely affect patients’ nutritional status and overall recovery. Recent retrospective analyses demonstrate that diarrhea is nearly universal in patients receiving HDM, whereas nausea is highly prevalent and vomiting occurs in approximately 60% of patients, with grade ≥2 diarrhea being especially common and linked to longer hospital stays and greater clinical burden (e.g., electrolyte imbalances and hypoalbuminemia) in this population [1].

In clinical practice, the dose of HDM used for conditioning varies according to the underlying hematological malignancy and patient-related factors. In patients with multiple myeloma, melphalan at a dose of 200 mg/m² is widely used as the standard conditioning regimen before autologous HSCT, as supported by clinical guidelines and contemporary reviews. In selected populations, particularly older or frail patients, reduced dosing (e.g., 140 mg/m²) may be considered to limit treatment-related toxicity [2-4]. In lymphoma, melphalan is commonly incorporated into the BEAM conditioning regimen (carmustine, etoposide, cytarabine, and melphalan), where it is typically administered at a dose of 140 mg/m² before stem cell infusion, as described in clinical protocols and contemporary reviews [5]. These high doses are associated with substantial mucosal and GI toxicity due to the cytotoxic effects of melphalan on rapidly dividing epithelial cells of the GI tract [1,6].

Oral mucositis is one of the most frequent and debilitating toxicities associated with HDM conditioning. Preventive supportive care measures are therefore routinely implemented in transplant units to reduce mucosal injury and maintain oral intake. Standard oral care protocols commonly include regular mouth rinses with sodium bicarbonate (sodium hydrogen carbonate) solutions, which help maintain oral pH and reduce mucosal irritation [7]. In addition, topical antifungal prophylaxis such as amphotericin B oral suspension (Fungizone, Ben Venue Laboratories, Inc., Bedford, OH) is often used to prevent secondary fungal colonization and infection of the damaged oral mucosa [8]. Oral cryotherapy has also been shown to reduce the incidence and severity of melphalan-induced oral mucositis and may indirectly help preserve oral intake. These preventive strategies, combined with meticulous oral hygiene and supportive care, aim to decrease the severity of mucositis, improve patient comfort, and help preserve nutritional intake during the vulnerable post‑conditioning period [7].

The integrity of the GI tract is essential for maintaining adequate nutrition, particularly during the physiologically intense period of HSCT. High-dose chemotherapy disrupts the integrity of the GI mucosa, leading to mucositis, nausea, vomiting, and diarrhea that markedly impair oral intake and nutrient absorption. These GI toxicities frequently emerge early after conditioning and reduce patients’ ability to ingest food, often dropping oral intake below estimated energy requirements within the first week of treatment. This decline in oral intake contributes to weight loss and depletion of protein and energy reserves. However, malnutrition in HSCT recipients is multifactorial and results from the interplay between reduced nutrient intake, systemic inflammation, prior treatment exposure, and the hypercatabolic state induced by chemotherapy, all of which contribute to nutritional deterioration [9].

Malnutrition is highly prevalent among patients undergoing HSCT [10]. Epidemiological studies suggest that approximately 10%-15% of patients present with pre-existing malnutrition before transplantation, while the prevalence may increase to 20%-30% during the post-transplant period as a consequence of treatment-related toxicities and reduced oral intake [11]. Furthermore, prospective evaluations using validated tools such as the Patient-Generated Subjective Global Assessment (PG-SGA) have reported that up to two-thirds of transplant recipients require some form of nutritional intervention during the early stages of transplantation [12]. These findings illustrate the substantial burden of nutritional deterioration in this population and emphasize the importance of systematic nutritional monitoring throughout the transplant process [9]. Importantly, many patients already present with nutritional vulnerability at the time of transplant admission, reflecting the cumulative effects of prior chemotherapy and disease-related metabolic alterations. Early identification of nutritional risk is therefore essential to prevent rapid deterioration during the conditioning phase.

Compounding this, the metabolic demands imposed by systemic inflammation and the catabolic effects of chemotherapy further accelerate muscle breakdown and loss of lean body mass. As a result, the balance between nutrient supply and metabolic needs becomes severely compromised, increasing the risk of electrolyte disturbances and metabolic derangements that require ongoing clinical management [9]. Importantly, malnutrition in the HSCT setting is increasingly recognized as a major determinant of adverse clinical outcomes rather than a simple secondary consequence of treatment. Patients with compromised nutritional status have been shown to experience higher rates of infectious complications, longer periods of hospitalization, and an overall greater burden of treatment-related morbidity. While some studies investigating specific survival endpoints have produced mixed results, trends toward increased infection rates and hospital readmissions among malnourished patients have been observed [13].

Despite strong evidence supporting the importance of nutrition in transplant care, routine nutritional assessment and early intervention remain inconsistently implemented across clinical settings. International guidelines from bodies such as the American Society for Parenteral and Enteral Nutrition (ASPEN) and the European Society for Clinical Nutrition and Metabolism (ESPEN) recommend systematic screening for malnutrition at admission and throughout the transplant process; however, these practices are not uniformly implemented across transplant centers worldwide [12]. Validated screening tools like the PG-SGA and other instruments such as the Subjective Global Assessment (SGA), Nutritional Risk Screening 2002 (NRS 2002), or the Malnutrition Universal Screening Tool (MUST) have been shown to identify patients at risk of malnutrition before and after HSCT [14-16]. For example, in one cohort of patients undergoing HSCT, the use of PG-SGA demonstrated that nutritional risk and malnutrition increased dramatically after transplant compared with pre-transplant status [11]. However, these tools are often underused, and systematic screening is not consistently integrated into clinical pathways, contributing to heterogeneity in supportive care practices [17].

The early engagement of clinical nutrition specialists, dietitians, and nutrition support teams is equally limited in many institutions. When nutritional risk is identified, a multidisciplinary approach is essential to tailor nutrition support strategies to individual needs, whether by optimizing oral intake, providing enteral nutrition when the GI tract is functional, or initiating parenteral nutrition when necessary [12].

Current nutritional guidelines also emphasize the importance of meeting adequate energy and protein requirements in patients undergoing HSCT. Expert recommendations from the ESPEN indicate that patients receiving intensive chemotherapy or transplantation generally require energy intake of approximately 25-30 kcal/kg/day and protein intake of 1.2-1.5 g/kg/day to counterbalance the hypercatabolic state induced by systemic inflammation and treatment-related stress. When oral intake becomes insufficient due to mucositis, nausea, vomiting, or diarrhea, nutritional support should be escalated to enteral or parenteral nutrition to prevent acute malnutrition and maintain metabolic stability [10].

Emerging evidence suggests that proactive nutritional care mitigates the severity of malnutrition and may improve clinical outcomes. In a prospective study, patients who received an oral polymeric formulation with tailored nutritional support according to PG-SGA scoring had significantly lower rates of severe malnutrition after transplant and demonstrated improved median overall survival compared with those who received inadequate nutritional support [18]. Additionally, integrated nutritional interventions have been shown to preserve anthropometric and biochemical markers of nutrition and reduce the proportion of patients classified as malnourished after HSCT [19]. Collectively, these data highlight that systematic nutritional screening, early specialist involvement, and individualized nutrition support are not merely supportive extras but critical components of transplant care that influence both short- and long-term patient outcomes. In conclusion, melphalan-related GI toxicity significantly jeopardizes nutritional status during the transplant period. Systematic nutritional screening, early intervention, and structured care pathways should be integrated into transplant units to optimize supportive care and improve patient outcomes. Future efforts should focus on implementing standardized nutritional pathways within transplant units and conducting prospective studies to better define the impact of tailored nutritional interventions on transplant outcomes.
